# Structural Dynamics of Troponin I during Ca2+-Activation of Cardiac Thin Filaments: A Multi-Site Förster Resonance Energy Transfer Study

**DOI:** 10.1371/journal.pone.0050420

**Published:** 2012-12-05

**Authors:** Hui Wang, Joseph M. Chalovich, Gerard Marriott

**Affiliations:** 1 Department of Pharmacology, School of Medicine, University of North Carolina, Chapel Hill, North Carolina, United States of America; 2 Department of Biochemistry and Molecular Biology, Brody School of Medicine at East Carolina University, Greenville, North Carolina, United States of America; 3 Department of Bioengineering, University of California, Berkeley, California, United States of America; University of Heidelberg Medical School, Germany

## Abstract

A multi-site, steady-state Förster resonance energy transfer (FRET) approach was used to quantify Ca^2+^-induced changes in proximity between donor loci on human cardiac troponin I (cTnI), and acceptor loci on human cardiac tropomyosin (cTm) and F-actin within functional thin filaments. A fluorescent donor probe was introduced to unique and key cysteine residues on the C- and N-termini of cTnI. A FRET acceptor probe was introduced to one of three sites located on the inner or outer domain of F-actin, namely Cys-374 and the phalloidin-binding site on F-actin, and Cys-190 of cTm. Unlike earlier FRET analyses of protein dynamics within the thin filament, this study considered the effects of non-random distribution of dipoles for the donor and acceptor probes. The major conclusion drawn from this study is that Ca^2+^ and myosin S1-binding to the thin filament results in movement of the C-terminal domain of cTnI from the outer domain of F-actin towards the inner domain, which is associated with the myosin-binding. A hinge-linkage model is used to best-describe the finding of a Ca^2+^-induced movement of the C-terminus of cTnI with a stationary N-terminus. This dynamic model of the activation of the thin filament is discussed in the context of other structural and biochemical studies on normal and mutant cTnI found in hypertrophic cardiomyopathies.

## Introduction

The Ca^2+^-tropinin complex acts as a molecular-switch in the regulation of cardiac muscle contraction. Cardiac thin filaments are composed of polymerized actin protomers decorated with stoichiometric levels of the tropomyosin dimer (cTm) and the troponin complex (cTn), in the ratio of 7∶1∶1 respectively. cTn is composed of three subunits: troponin C (TnC), troponin I (TnI) and troponin T (TnT) [1]. TnC, which serves as the Ca^2+^-receptor, has two globular sub-domains linked by an extended α-helix [2], [3]. TnI interacts with F-actin, TnC, TnT and tropomyosin (cTm). TnI inhibits the actomyosin ATPase in the thin filament although inhibition is reversed upon Ca^2+^-binding to the TnC complex [4]. High-resolution structural analyses have shown that the two EF-hand motifs on the N-lobe of skeletal TnC open up upon Ca^2+^-binding [5]–[8] leading to the unfolding of a short α-helix within the TnI inhibitory segment [9]. This conformational change allows TnI to bind more tightly to TnC [10], [11] and, as a result, the interaction between TnI and F-actin weakens. This latter change is key to the activation of the thin filament, as it should allow myosin to form a strong bond with F-actin [12], [13]. Collectively, these studies suggest that the activation of the thin filament requires significant, Ca^2+^-triggered, concerted structural dynamics [14].

Understanding the role of protein structural dynamics in Ca^2+^-regulation of muscle contraction might help to explain how specific single point mutations in thin filament proteins lead to hypertrophic and dilated cardiomyopathies (HCM and DCM). For example, many of the HCM mutations in TnC and TnI that lead to reduced cardiac output are single point and conservative [15]–[19], and on the face of it, unlikely to alter the overall structure or interactions of the mutated protein within the thin filament. We are testing the hypothesis that the deleterious effects of these mutations are a result of altered structural dynamics, for example one that might change the rate of a conformational transition or coupling to a neighboring subunit in the filament. Ideally one would test this hypothesis by carrying out high-resolution structural analyses of individual Tn subunits within functional thin filaments at different states of the crossbridge cycle. Cryo-electron microscopy of reconstituted or intact muscle fibers is the most informative of these techniques although the information is carried out on fixed samples. On the other hand, Förster resonance energy transfer (FRET) provides sensitive and dynamic information on changes in proximity between specific loci on multiple labeled proteins within functional thin filament under physiological conditions of temperature and ionic composition. The FRET-approach has been applied in the characterization of proximity relationships within or between Tn subunits and their complexes with F-actin. Some of these studies indicate that Ca^2+^-binding to TnC brings about a change of conformation in the region of the TnI-TnC interface [20]–[24]. For example, in fast-skeletal (fs) TnI, Cys-133 is thought to move away from sites on actin including Gln-41, Lys-61, Cys-374 and the bound nucleotide, while a far smaller change is found at the N-terminus of skTnI (Cys-9). These studies indicate that the Ca^2+^-triggered activation of the thin filament may proceed in part via a hinge-like bending motion of TnI, in which the C-terminal domain of TnI moves away from the outer domain of actin filament while the N-terminal domain of TnI remains fixed [25], [26]. Farah, et al. [27] also showed that the C-terminal region of fsTnI (at least from residue 103 to 156) is involved in the Ca^2+^-dependent regulation of the thin filament. Finally a recent kinetic study suggests that structural transitions are involved during two steps of Ca^2+^-activation of the thin filament – these include the rapid dissociation of the C-terminal (mobile) domain of TnI from the actin filament, associated with Ca^2+^-binding, and a slower switching of the inhibitory region on TnI that is implicated in inhibiting the formation of strongly bound cross-bridges [28].

**Figure 1:**
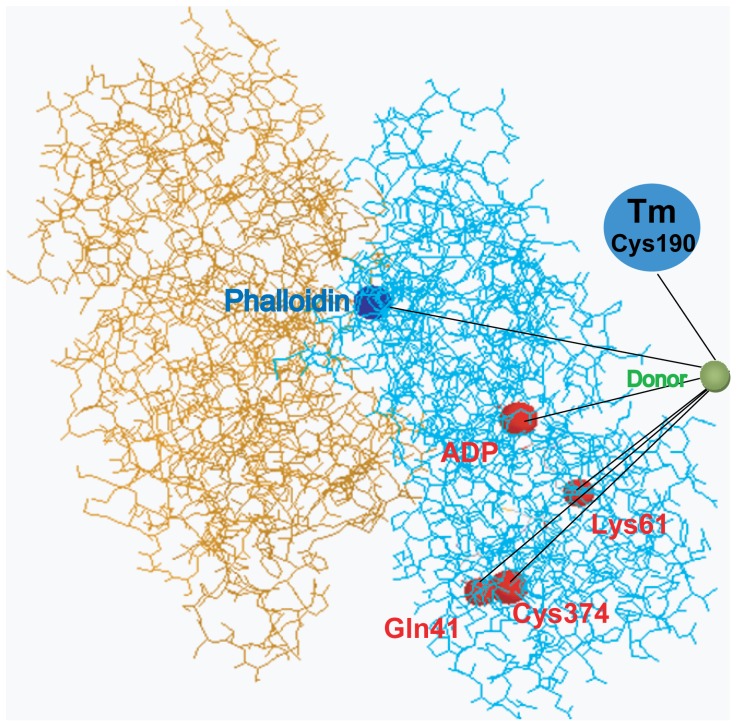
A cross section of the thin filament based on an actin filament model by Oda et al. [45]. The acceptor labeling sties used in previous FRET studies (Gln41, Lys61, Cys374 and the bound nucleotide) are shown as red balls, and are located in the outer domain of actin filament. In this study, acceptors are labeled on phalloidin (shown as a blue ball on the inner domain of actin filament), Cys374 on actin, or Cys190 on cTm. The donor probe labeled on cTnI is shown as a green ball.

These earlier studies have for the most part been carried out on thin filament preparations derived from skeletal muscle. Now while the component proteins of cardiac thin filaments are homologous to those in skeletal muscle, there are some significant differences in their structure and mode of regulation. For example, cTnI has 31 additional residues at the N-terminus and phosphorylation within this region decreases the Ca^2+^ sensitivity of the cardiac thin filament [29], [30]. Moreover, earlier FRET-based analysis of Ca^2+^-mediated changes in proximity between sites on TnI and F-actin have been limited to labeling sites on the outer domain of F-actin (Figure 1). Moreover, the analysis of FRET efficiency in these studies were based on random distributions of dipole moments for the donor and acceptor probes, which is not valid when one or both probes are immobile during the excited state lifetime [14], [31].

We improved and extended these earlier FRET studies in an investigation of the nature of conformational transitions within human cTnI during Ca^2+^-activation of contraction within fully functional, recostituted thin filaments. The multi-site FRET analysis includes probing proximity changes in response to Ca^2+^- and myosin II binding to thin filaments and an evaluation of the effect of the orientation of donor and acceptor dipole moments on calculated changes in proximity [14]. The donor sites on cTnI were chosen to minimize any structral perturbation associated with their labelling, and included residues 150–186 of cTnI, which inhibits actomyosin ATPase [27] and is known to move away from Cys-374 of F-actin. The acceptor sites were selected to probe motions of cTnO with respect to the outer and inner domains of F-actin as well as cTm.

## Materials and Methods

### Cardiac tropomyosin and troponin I

The cDNA of human cardiac tropomyosin and troponin I were isolated by PCR from an adult human heart cDNA library (Invitrogen) with the following primers:

For cTm: GCGGGATCCATGGACGCCATCAAGAAGAAGATG (Forward),

GCGAAGCTTTTATATGGAAGTCATATCGTTGAGAG (Reverse)

For cTnI: GCGGGATCCATGGCGGATGGGAGCAGCG (Forward),

GCGAAGCTTTCAGCTCTCAAACTTTTTCTTGC (Reverse),

The endogenous cysteine residues in wild type cTnI (Cys80 and Cys 97) were substituted with serine. Other substitutions in cTnI were made at V107C, A151C, V188C, A197C and S210C to generate five single cysteine mutants. The genes of cTnI mutants and cTm were cloned into vector pQE-30(Qiagen, CA) between BamH I and Hind III sites. The proteins were expressed in M15pRep4 E. coli strain and purified by running through Ni-NTA column (Qiagen, CA) in the presence of 6 M urea.

### Other protein preparation

Chicken skeletal G-actin was prepared from acetone powder using the standard polymerization/depolymerization protocol as described by Heidecker et al [32]. Bovine cTnT and cTnC are prepared according to Potter [33]. Rabbit skeletal myosin S1 was prepared according to Weeds & Taylor [34].

### Labeling of proteins

Cysteine residues in G-actin and cTm were labeled in the presence of a 5-fold excess of TMR-5-maleimide (Invitrogen) while cTnI mutants were labeled using a 5-fold excess of fluorescein-5-maleimide (Anaspec) in DTT-free G-buffer (5 mM Tris, 0.2 mM CaCl_2_, 0.1 mM ATP, pH 8.0), Tm buffer (50 mM Tris, 500 mM KCl, pH8.0), or urea buffer (50 mM Tris, 500 mM KCl, 6 M urea, pH8.0) respectively at 37°C for 2 hours. Unlabeled dye was removed by running the reaction mixture through an Econo-Pac 10DG column (Bio-Rad) for G-actin and cTm or a Ni-NTA column for cTnI. SDS-PAGE was used to confirm all of the dye was covalently bound to the protein. The final concentration of the fluorescence label on the protein was determined from measurements of their absorption spectra recorded on a Shimadzu PC1601 spectrophotometer. The corresponding concentration of protein in the conjugate was measured by using the Bradford protein assay kit (Bio-Rad). The labeling ratios were calculated by dividing the concentration of the fluorescence probe by that of the protein.

### Other reagents

Prof. Heinz Faulstich, Max Planck Institute for Cell Biology kindly provided IC3-Phalloidin for these studies.

### Reconstitution of thin filaments

Each of the 5 single cysteine labeled cTnI mutants (Fluorescein-cTnI) were mixed with purified bovine cardiac cTnT and cTnC at a molar ratio of 1∶1∶1 in urea buffer and dialyzed against buffers containing 6 M, 4 M, 3 M, 2 M, 1 M and 0 M urea in order to reconstitute the functional cTn complex. Superose 6 chromatography was used to purify labeled cTn complexes. TMR-labeled and unlabeled G-actin were polymerized overnight at 4C in the presence of a 1.5-fold excess of IC3-phalloidin or unlabeled phalloidin in F-buffer (0.2 mM CaCl_2_, 0.1 mM ATP, 1 mM DTT, 100 mM KCl, 2 mM MgCl_2_ and 5 mM Tris, pH 8.0). The F-actin complex was purified by centrifugation at 85,000× g for an hour and by resuspending the pellet in F-buffer. Thin filaments were reconstituted with F-actin, cTm and cTn with a molar ratio of 7∶1∶1. The mixture was left on ice overnight to allow for complex formation and then centrifuged at 85,000× g for an hour. The pellet was resuspended in ATPase assay buffer (10 mM MOPS, 3 mM MgCl_2_ and 1 mM ATP, pH 7.0, with 0.1 mM CaCl_2_ or 1 mM EGTA) for the ATPase assays, or in F-buffer for fluorescence measurement.

### ATPase activity assays

The malachite green phosphate assay was used to measure the Ca^2+^-activated S1-ATPase activity in thin filaments [14], [35].

### Fluorescence spectra

Steady state emission spectra were measured at 25°C using an SLM-AB2 fluorimeter, with excitation wavelength at 490 nm and emission between 500∼650 nm (both with 4 nm band pass). The magic angle excitation was employed to eliminate polarization errors from the instrument [36].

### FRET based molecular proximity analysis

The efficiency of FRET between a unique donor and a unique acceptor probe in the thin filament was calculated using the relationship as detailed in Wang et al [14]:
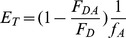
where F_DA_ and F_D_ are the quantum yields of the donor probe in the presence and absence of the acceptor probes respectively and f_A_ is the fraction of acceptor labeling. The efficiency of FRET (E_T_) is related to the distance between the donor probe (R) and the acceptor probes according to:
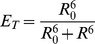
where R_0_ is the Förster distance, which is given by Lakowicz [37]

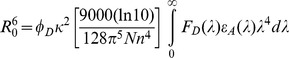



For an assumed κ^2^ value of 2/3, and with *n* being taken 1.33, then by expressing F(λ) in units of M^−1^cm^−1^(nm)^4^, the Förster distance (in Å) can be calculated by using:
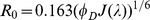



Where J(λ) is the spectral overlap integral, which can be calculated using the expression
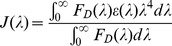



## Results

### Design of FRET experiments in reconstituted cardiac thin filaments

Farah and Reinach [27] showed that residues 120–156 of skTnI (corresponding to residue 150–186 of cTnI) are essential for the inhibitory function that skTnI exerts on the thin filament. In this study five sites within this region of cTnI were selected for donor labeling corresponding to residue numbers 107, 151, 188, 197 and 210,(see later figures of the thin filament structure). The fixed acceptor sites included those on the outer and inner domains of F-actin and at Cys-190 of cTm. All eight labeling sites were chosen so as to minimize the impact of the probe molecule and in replacing a wild-type residue with a cysteine residue. The donor and acceptor sites employed in this study greatly expands the scope of FRET-based determinations of proximity in relaxed and activated thin filaments compared to earlier studies.

### Labeling of cTnI with Fluorescein-5-maleimide

The labeling ratio of fluorescein to cTnI in the five cTnI mutants was calculated by using absorption spectroscopy and the Bradford assay to measure the concentrations of fluorescein and cTnI respectively [32]. The calculated labeling ratio was close to parity in each of the 5 conjugates: 0.83 for TnI 107C; 0.87 for TnI 151C; 0.83 for TnI 188C; 0.92 for TnI 197C; and 0.87 for TnI 210C.

**Figure 2:**
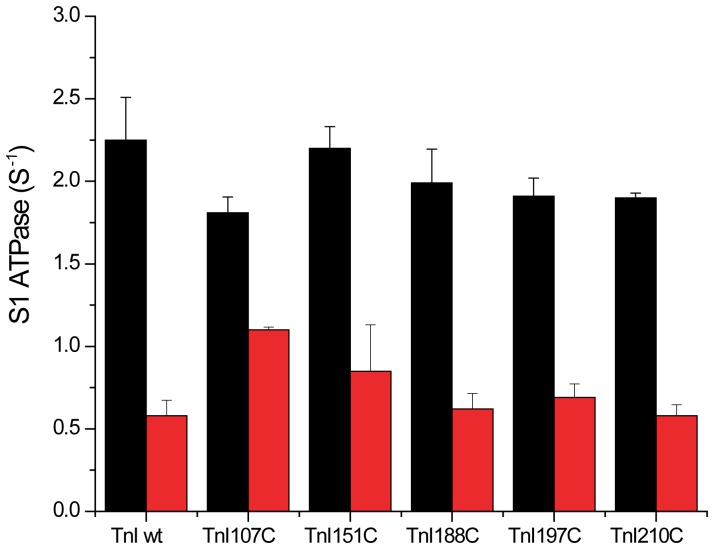
The Ca^2+^-regulated S1 ATPase activities within thin filaments reconstituted with wild-type cTn and cTn mutants conjugates. Fluorescein labeled human cTnI single cysteine mutants were reconstituted into cTn complex with bovine cTnC and cTnT as described in the methods section. 0.71 µM wild-type bovine cTn or reconstituted cTn was mixed with 0.71 µM human cTm and 5 µM chicken skeletal actin filament to reconstitute thin filaments. 0.1 µM S1 was added to the thin filaments and their ATPase activities were measured at 25°C in ATPase assay buffer (10 mM MOPS, 3 mM MgCl_2_ and 1 mM ATP, pH 7.0, with 0.1 mM CaCl_2_ or 1 mM EGTA). The ATPase activities were measured by determining the free phosphate concentration using the Malachite green method. The ATPase rates are shown as columns (n = 3) for the wild-type cTn and cTn mutants conjugates (i.e. cTn107C, cTn151C, cTn188C, cTn197C and cTn210C), in which the color scheme is black for the thin filament in the presence of Ca^2+^ and red without Ca^2+^.

The effect of introducing a fluorescein group into the cTnI on the function and regulation of the reconstituted thin filament was evaluated for each mutant by measuring the S1-ATPase in the absence and presence of calcium. A thin filament preparation reconstituted with unlabeled wild type bovine cTn was used as a control sample for these studies (Figure 2). In most cases, the reconstituted fluorescein-cTnI labeled thin filaments exhibited rates of ATPase activity in the absence and presence of Ca^2+^-that were similar to the respective rates with control thin filaments. In the case of TnI107C, the activation by Ca^2+^ was somewhat reduced.

### FRET based analysis the cTnI mutants conjugates within thin filaments

Having shown that cTnI conjugates of fluorescein can substitute for unlabelled cTnI within functional, reconstituted thin filaments, steady-state fluorescence emission spectra were recorded for each thin filament preparation in the absence and presence of acceptor probes. These studies allow for the calculation of the quantum yield of the fluorescein-cTnI donor probe in each preparation, and the efficiency of FRET. The quantum yield of the fluorescein in each sample was determined according to:
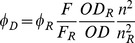
where *Φ*_R_ is the fluorescence intensity of a dilute solution (OD<0.1 at 480 nm) of fluorescein in 0.1 M NaOH, which has a known quantum yield taken as 0.95 [37]. F and F_R_ are the integrated fluorescence emission intensities of fluorescein in the thin filament preparation and in the reference solution respectively; OD and OD_R_ are the optical densities (absorption at 490 nm) of fluorescein the thin filament preparation and in the reference solution respectively*; n* and *n*_R_ are the refractive indices of the thin filament preparation and the reference respectively, which are taken as being identical at 1.33. Fluorescence spectral measurements were conducted at 22°C using an excitation wavelength of 490 nm, while the emission intensity was integrated between 500 nm and 650 nm. The values of the relative quantum yields were calculated as indicated and are summarized in Table 1.

**Table 1:** Quantum yields of Fluorescein labeled troponin I single cysteine mutants and Forster distances between Fluorescein and the acceptors in different conditions.

	Quantum yields				Foerster distances (nm)							
					Fluorescein-TMR				Fluorescein-IC3			
	−S1		+S1		−S1		+S1		−S1		+S1	
	−Ca^2+^	+Ca^2+^	−Ca^2+^	+Ca^2+^	−Ca^2+^	+Ca^2+^	−Ca^2+^	+Ca^2+^	−Ca^2+^	+Ca^2+^	−Ca^2+^	+Ca^2+^
TnI107	0.26	0.29	0.29	0.28	4.4	4.5	4.5	4.4	4.8	4.9	4.9	4.8
TnI151	0.30	0.32	0.34	0.35	4.5	4. 6	4.6	4.6	4.9	5.0	5.0	5.0
TnI188	0.31	0.35	0.36	0.38	4.5	4.6	4.6	4.7	4.9	5.0	5.1	5.1
TnI197	0.33	0.35	0.34	0.38	4.6	4.6	4.6	4.7	5.0	5.0	5.0	5.1
TnI210	0.32	0.32	0.32	0.32	4.5	4.6	4.5	4.6	5.0	5.0	5.0	5.0

### FRET analysis and the problem of the orientation factor

The overlap integral between the absorption spectrum of the acceptor probe and the emission spectrum of the donor probe, J(λ), was calculated according to Wang et al (2006) using a molar extinction coefficient for Cy3 of 150,000 M^−1^ cm^−1^ (GE Healthcare) and 95,000 M^−1^ cm^−1^ for TMR (Invitrogen). The results of these calculations are: for the fluorescein-IC3 FRET pair J(λ) = 2.488×10^15^ M^−1^ cm^−1^(nm)^4^ and for the fluorescein-TMR FRET pair, J(λ) = 1.476×10^15^ M^−1^ cm^−1^(nm)^4^. The only unknown parameter in the Förster equation is the orientation factor,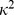, which can be computed using the relationship:
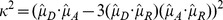
where 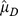, 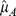, 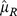 are the unit vectors of the donor dipole moment, the acceptor dipole moment and the vector connecting the centers of the donor and acceptor. One needs to know the coordinates of the donor and acceptor dipoles in order to calculate the orientation factor, and these are rarely available (but see Yan & Marriott [38] for an experimental determination of *κ* ^2^). However, if one can show that both probes undergo unrestricted, isotropic motions during their excited state lifetime, then one is justified in using an averaged orientation factor 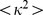 of 2/3 in the FRET calculation. Orientational freedom is widely assumed and applied to distance calculations using FRET often without any justification e.g. by measuring the steady-state anisotropy values of the donor and acceptor probes. Moreover, Vanbeek et al [31] have argued that this assumption is not strictly valid in cases where the orientation and the distance between the donor and acceptor dipoles are correlated. On the other hand, Muñoz-Losa, et al [39]. found that the error introduced by using an ideal dipole approximation decreased while the distance increased. The error drops to <10% for distances greater than 5 nm (i.e. >R_0_).

The *κ* ^2^ value can be estimated by using the Dale-Eisinger approach [14], [40], [41], which assumes that one dipole is fixed while the other one undergoes an isotropic motion i.e.,
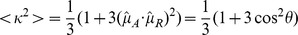
where θ is the angle between the acceptor dipole moment and the line connecting the centers of the donor and acceptor. The largest error introduced by using this ideal dipole approximation should not exceed 12% [14]. From Table 2, it can be seen that the steady-state anisotropy values for the five fluorescein conjugates of cTnI, in reconstituted thin filaments, were low (∼0.2) compared to values for the corresponding acceptor probes (>0.28). Thus the assumption that the dipoles of the donor and acceptor probes randomize during their excited state lifetimes is not justified. To assess the effect of the limited distribution of acceptor dipole orientation expected from the high anisotropy value, we used the high resolution structure of TMR-G-actin [42] (PDB: 1J6Z) and a published EM model of skeletal thin filament [43] to estimate the angle θ of the acceptor (TMR) probe on actin with regards to the filament axis. As shown in Table 3, the difference in the calculation of the Förster distance assuming the ideal dipole approximation from that calculated from the model of the thin filament is <9% for residues 74 and 118 of sTnI, corresponding to residues 107 and 151 in cTnI. The Pirani structure [43] is based on the model of F-actin filament constructed by Holmes [44], which is slightly different from the more recent model [45] in terms of the orientation of the actin protomer. In spite of this difference, according to the previous argument the error in the distance calculation using the ideal dipole approximation cannot exceed a value of 12%. Thus, in spite of the polarized emission of the donor and acceptor probes in the thin filament preparations, we are nonetheless justified in using the ideal dipole approximation i.e. 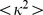 of 2/3 in our calculations. The values of R_0_ for the donor-acceptor probes used in this study are summarized in Table 1.

**Table 2:** Anisotropy of the donor and acceptor probes labeled in the thin filaments reconstituted with wild-type or single cysteine mutants of cTnI.

	Donor					Acceptor		
	TnI107	TnI151	TnI188	TnI197	TnI210	IC3-ph	actin374	Tm190
−Ca^2+^	0.203	0.176	0.200	0.203	0.172	0.285	0.313	0.242
+Ca^2+^	0.225	0.212	0.203	0.180	0.154	0.283	0.320	0.245
+Ca^2+^,+S1	0.216	0.226	0.190	0.186	0.157	0.293	0.313	0.281

**Table 3:** Orientation of TMR labeled on cys-374 on actin to skeletal TnI residues calculated from the thin filament model (Pirani, *et al.*, 2006), and its effect on Foster distance calculation.

	sTnI74(−Ca^2+^/+Ca^2+^)	sTnI118(−Ca^2+^)	sTnI118(+Ca^2+^)
cos^2^θ	0.037	0.118	0.666
<κ^2^>	0.370	0.451	0.999
R_0_/R_0_ (2/3)	0.91	0.94	1.07

**Figure 3:**
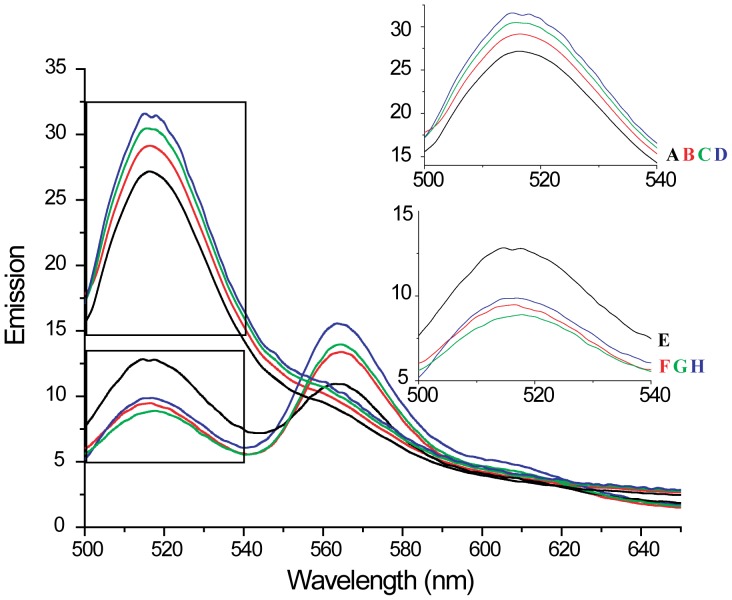
Emission spectra of the thin filament reconstituted with Fluorescein labeled cTnI151C in the presence of unlabeled- and IC3-labeled phalloidin. Fluorescein labeled cTnI151C was reconstituted into cTn with bovine cTnC and cTnT. 0.71 µM reconstituted cTn was mixed with 0.71 µM human cTm and 5 µM actin filament in the presence of unlabeled- and IC3-labeled phalloidin to reconstitute the functional thin filaments. Emission spectra were recorded at 25°C in F-buffer with the excitation wavelength of 490 nm and emission wavelengths from 500 nm to 750 nm. where: (A) Tn/Tm/F-actin/phalloidin/-Ca^2+^, (B) Tn/Tm/F-actin/phalloidin/+Ca^2+^, (C) Tn/Tm/F-actin/phalloidin/S1/-Ca^2+^, (D) Tn/Tm/F-actin/phalloidin/S1/+Ca^2+^, (E) Tn/Tm/F-actin/IC3-phalloidin/-Ca^2+^, (F) Tn/Tm/F-actin/IC3-phalloidin/+Ca^2+^, (G) Tn/Tm/F-actin/IC3-phalloidin/S1/-Ca^2+^, (H) Tn/Tm/F-actin/IC3-phalloidin/S1/+Ca^2+^.

The emission spectrum of fluorescein labeled cTnI (151C) exhibited a maximum intensity at 515 nm in the absence of acceptor. The intensity at this wavelength increased upon Ca^2+^/S1 binding to the thin filament, as shown in figure 3. In the presence of the acceptor (IC3-phalloidin), the fluorescence intensity of the donor at 515 nm decreased with concomitant appearance of a sensitized emission from the acceptor probe at 565 nm. The large degree of quenching of the donor emission at 515 nm made it possible to accurately determine the FRET efficiency and to carry out determinations of the distance between the donor and acceptor probes in the absence and presence of calcium and myosin, using the equations described in Materials and Methods and the R_0_ values calculated in Table 1. These measurements and calculations were repeated for each of the 5 cTnI donor probes and acceptor sites on F-actin and cTm.

### Conformational change of cTnI upon Ca^2+^/S1 binding to the thin filaments

**Figure 4:**
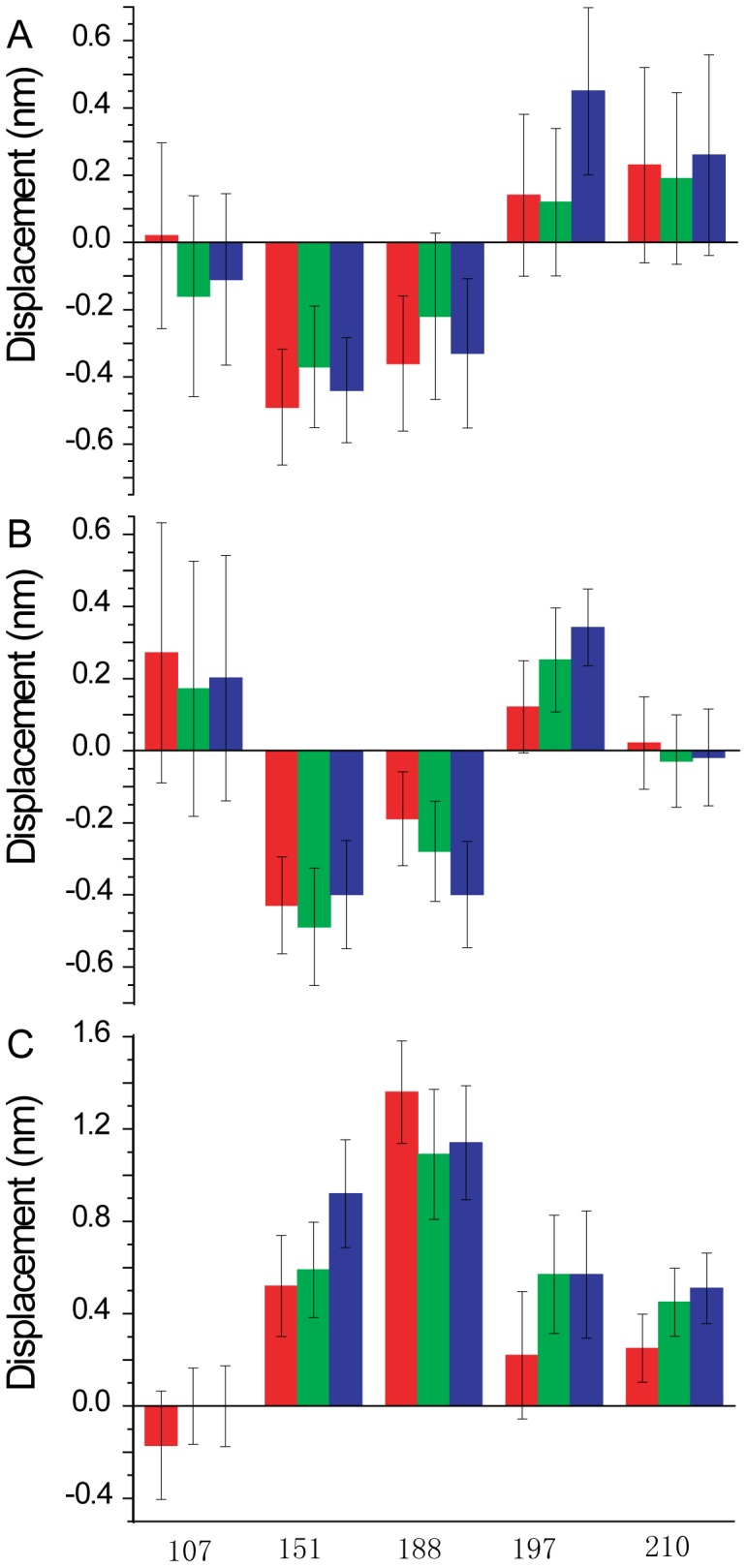
Relative displacements of donor sites on cTnI to acceptor sites within the thin filament upon Ca^2+^ and/or S1 binding. The distances were obtained by subtracting the distances between donors and acceptors within the thin filament in the relaxed state from that in the presence of Ca^2+^ and/or S1. Positive values indicate that donors move away from acceptors and negative values indicate that donors move towards acceptors. The color scheme is red for the displacements of the donor on cTnI upon Ca^2+^-binding, green for that upon S1-binding and blue for that upon both Ca^2+^ and S1-binding. (A) Relative displacements of Fluorescein on sites of cTnI to IC3 on phalloidin. (B) Relative displacements of Fluorescein on sites of cTnI to TMR on Cys190 on cTm. (C) Relative displacements of Fluorescein on sites of cTnI to TMR on Cys374 on actin.

A matrix of the calculated FRET distances between the donor and acceptor probes in the relaxed state of the thin filament are shown in table 4, with the corresponding displacements of the donor probes shown schematically in figure 4. In particular, the binding of Ca^2+^ or S1 to the thin filament did not lead to any significant displacement of residue 107 on cTnI with respect to sites on F-actin. On the other hand, residues 151 and 188 of cTnI displayed consistent behaviors, i.e., they both underwent significant movements away from the outer domain of F-actin (Cysteine 374 of F-actin) by as much as 5 Å for cTnI151 and 13.6 Å for cTnI188. In both cases these residues moved toward the phalloidin binding site on the inner domain of the F-actin filament (4 Å for cTnI151 and 1.9 Å for cTnI188). These two residues also moved toward cysteine-190 on cTm (5 Å for cTnI151 and 3.6 Å for cTnI188). Residues 197 and 210 on cTnI moved away from all three acceptor sites, although residue 210 did not move very far from the Cysteine 190 of cTm. S1-binding did not induce any further movement of these sites compared to their Ca^2+^-activated loci. S1-binding alone however, resulted in similar movements to Ca^2+^ for relaxed thin filaments.

**Table 4:** Calculated distances between Donor labeled troponin I single cysteine mutants and acceptor labeled on actin/tropomyosin in the relaxed state (nm).

	TnI107	TnI151	TnI188	TnI197	TnI210
Phalloidin	5.2±0.3	4.8±0.1	4.7±0.1	4.8±0.1	4.6±0.1
Tm190	5.6**±**0.2	5.2±0.1	5.1±0.2	5.1±0.2	4.9±0.2
Actin374	5.4**±**0.1	4.5±0.2	4.8±0.1	4.3±0.2	4.3±0.1

## Discussion

**Figure 5:**
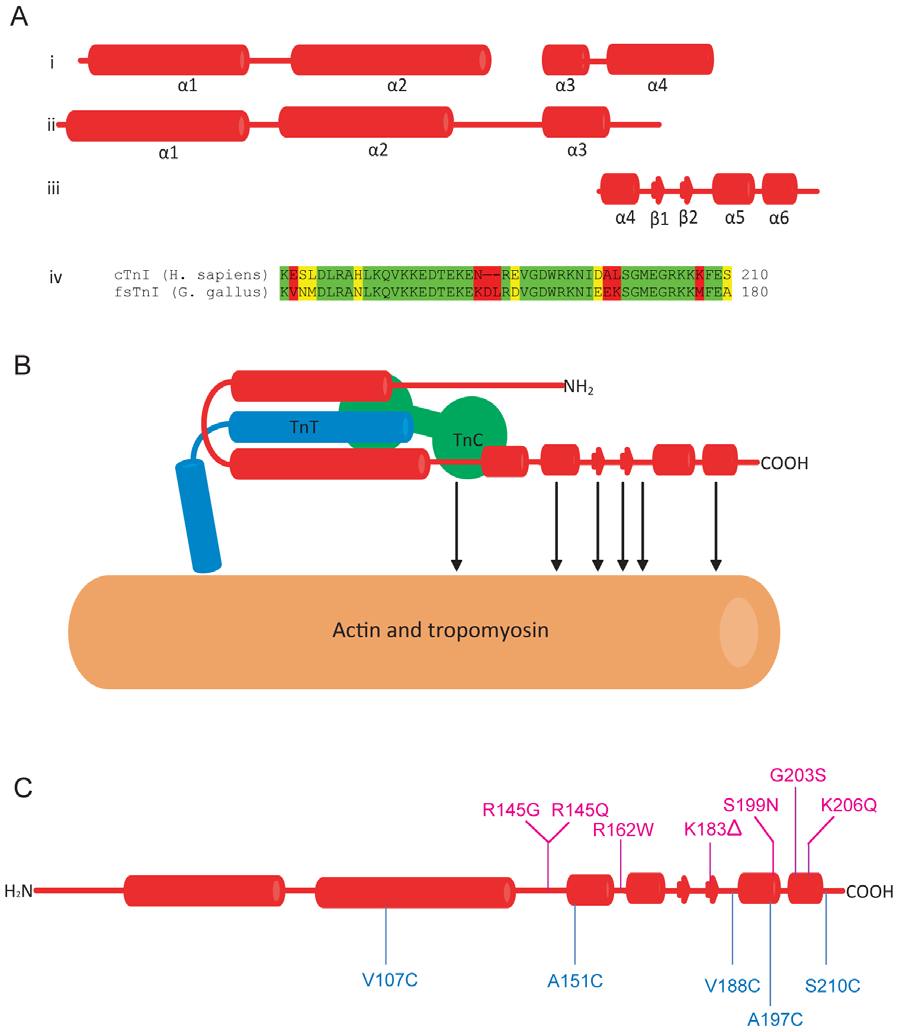
Schematic models of TnI. (A) structures of TnI. (i) Crystal structure of cTnI in the 52 kD core domain of cTn in the presence of Ca^2+^ (Takeda, et al., 2003; PDB 1J1E). Residues from 40 to 191 are resolved in this sturcture, and form four α-helices (α1, α2, α3 and α4). Helices α2 and α3 are linked by a flexible linker (residues 137–147) which is not resolved in the structure. (ii) Crystal structure of sTnI in the core domain of sTn in the presence of Ca^2+^ (Vinogradova, et al., 2005; PDB 1YTZ). Residues from 3–143 are resolved in this structure, which is very similar to the structure of cTnI. (iii) NMR stucture of C-terminus of sTnI [47] (PDB 1VDJ). Unlike the crystal structue of cTnI, the helix of α4 is much shorter, and was followed with a β-loop-β motif and two short α-helices (α5 and α6). (iv), Alignment of the mobile domains from human cTnI and chicken fsTnI, which are highly conserved. (B) Schematic structure of the calcium saturated thin filament. Helices α1 and α2 of cTnI interact with cTnT and form the “IT-arm”, which binds to the C-lobe of cTnC. N-lobe of cTnC interacts with cTnI helix α3 and the linker between α2 and α3. There are several potential actin binding sites on C-terminus domain of TnI [47], as indicated by arrows. The color scheme is red for cTnI, green for cTnC, blue for cTnT and orange for actin filament with cTm. (C) Point mutations that caused HCM (R145G/R145Q, R162W, K183Δ, S199N, G203S and K206Q) are showed in mengeta and the donor labeling sites on cTnI used in this study (residues 107, 151,188, 197 and 210) are showed in blue.

In a crystal structure of the 52 kD core domain of human cardiac Tn [46], the N-terminal region of TnI (residues 42–136), TnT (residues 203–271) and C-terminal domain of TnC constitute an “IT arm”, that anchors the complex at the C-terminus of Tm. Residues 150–159 of TnI form an α-helix (α3) that interacts with the N-terminal domain of TnC, whereas the adjacent α-helix (α4) on TnI between residues 164–188 does not interact with other components. These α-helices are linked to the IT arm by a flexible region, which is unresolved in the Takeda structure [46] The crystal structure of skeletal troponin [9] is similar to the cardiac complex, although skeletal Tn differs at the C-terminus of TnI (as shown in Figure 5A) [47]. In skTnI, the α4 helix is much shorter, and is followed by a β-loop-β motif and two short α-helices (α5 and α6). This region, the so-called the “mobile domain”, is highly conserved and has several potential F-actin binding sites (as shown in Figure 5A) [47]. The C-terminal region of cardiac TnI is also required for full inhibition of the actomyosin ATPase [48].

**Figure 6:**
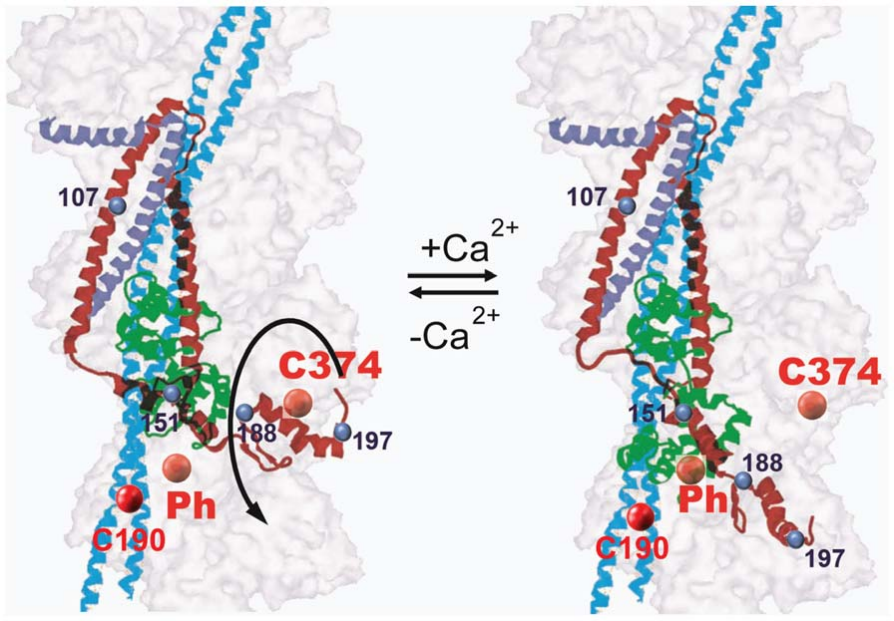
A Schematic model of Ca^2+^-induced troponin I movement on the thin filament. The binding of Ca^2+^ and interaction of cTnI triggers an opening of the N-lobe of cTnC, exposing a large hydrophobic site that increases the probability of interacting with cTnI. The negative free energy associated with this change drives a conformational change in cTnI, and a movement of the C-terminus domain cTnI away from the outer domain of actin filament. The color scheme is red for cTnI, green for cTnC, dark blue for cTnT and cyan for cTm. The actin filament is shown as transparent surface. Cysteine 374 and phalloidin binding site are shown as red balls and the donor labeling sites on cTnI are shown as blue balls.

Our study suggests that Ca^2+^ binding to TnC induces a movement of the C-terminal domain of TnI but there did not appear to be changes within regions of the IT arm (Figure 6). Helices α3 (indicated by residue 151) and β-loop-β motif (indicated by residue 188) move away from the outer domain of the actin filament and towards to the inner domain, while helices α5 and α6 (residue 197 and 210) move away from the filament. Previous FRET studies on skeletal TnI also showed that the helix α4 (indicated by residue 133) moves away from outer domain of actin filament upon Ca^2+^ binding, while helix α1 (indicated by residue 9) only moves a short distance [21]. These results prompted the idea that TnI behaves as a lever upon Ca^2+^ binding. In particular, while the lever head (IT arm) is somewhat stationary, the lever arm (α3, α4 and β-loop-β) undergoes an azimuthal movement from the outer domain of F-actin towards the inner domain of the filament. This movement induces a longitudinal movement of the C-terminus region (α5 and α6) away from the actin filament. Our study does not reveal any significant displacement between residue 210 on cTnI and the phalloidin binding site. This might due to the special geometry between the donor and acceptor sites in our system. Probes at position 107 of TnI, within the IT arm, were relatively unresponsive to Ca^2+^ or S1 binding. Modification at this position reduced the ability of troponin to inhibit the ATPase activity in the absence of Ca^2+^ (Fig. 2). It is possible that distance changes at this position were muted because the change in ATPase activity was also reduced. However, the degree to which the FRET changed was greater than the degree to which regulation differed from the other labeling positions. The most likely explanation is that the IT arm did not change or else that it changed in a relatively small degree.

Studies on dysfunctional forms of cTnI associated with hypertrophic cardiomyopathy (HCM) [15]–[17], [19] reveal that some of the disease-associated mutations localize to the C-terminus of cTnI and include residues 145, 162, 183, 199, 203 and 206 (as shown in figure 5C). These mutations alter the Ca^2+^-sensitivity of the cardiac thin filament [16], [17]. It is significant that our study shows that this region undergoes a large Ca^2+^-induced movement during activation of the thin filament.

In the steric-blocking and three-state models of thin filament activation [49], [50], Ca^2+^-binding is thought to induce a movement of tropomyosin on the thin filament. Although structural studies using X-ray diffraction and electron microscopy support this hypothesis [49], [51], [52], the majority of FRET studies do not show any evidence for such a movement [14], [53]–[56]. Our present study confirms this finding. According to our previous argument [14], cTm experiences thermally-driven fluctuations in the blocked and Ca^2+^-bound states of the thin filament that serve to decrease the average distance between the donor probe on cTm and a fixed acceptor probe on F-actin, to the point where FRET cannot accurately detect a change in the loci on adding calcium or myosin [14]. In the case of cTnI however, multi-site FRET analysis reveals that Ca^2+^ binding to the thin filament triggers a significant conformational transition for a specific domain of cTnI. This movement is consistent with the suggestion that Ca^2+^-induced azimuthal displacement of cTnI exposes a high affinity-binding site on the filament for S1 binding (S1-ADP or nucleotide-free S1). This conformational change may promote cooperative binding of additional S1 molecules to the thin filament, an event that has already been observed in the absence of ATP [57].

As we argued earlier [14], the binding of multiple S1 molecules to the thin filament may simply push the cTm molecule away from its locus in resting state on SD1 on F-actin and closer to sub-domain 3 (SD1), defining the open-state of the thin filament. The cooperative binding of S1 to the thin filament may also induce a movement of the inhibitory region of the cTnI that could be related to a slow kinetic process identified by Xing et al [28]. Finally, the cooperative binding of ATP-free S1 to the thin filament is also known to be dependent on a site within the 14 C-terminal residues of cTnT [58]. Deletion of these residues in cTnT prevents full inactivation of the thin filament in the absence of Ca^2+^ [59]. The relationship between changes in C-terminal TnT binding and the location of the C-terminal region of TnI binding shown here is unknown, although a multi-site FRET approach, as employed here, could shed light on the structural and/or functional significance of this observation.
